# Caveolae control the anti-inflammatory phenotype of senescent endothelial cells

**DOI:** 10.1111/acel.12270

**Published:** 2014-11-19

**Authors:** Elizabeth E Powter, Paul R Coleman, Mai H Tran, Angelina J Lay, Patrick Bertolino, Robert G Parton, Mathew A Vadas, Jennifer R Gamble

**Affiliations:** 1 Centre for the Endothelium, Vascular Biology Program, Centenary InstituteSydney, Australia; 2 Liver Immunology Group, Centenary InstituteSydney, Australia; 3 Institute for Molecular Bioscience and Centre for Microscopy and Microanalysis, The University of Queensland, University of St. LuciaQld, 4072, Australia; 4 The University of SydneyNSW, 2006, Australia

**Keywords:** ARHGAP18, caveolae, cellular senescence, endothelial cells, inflammation

## Abstract

Senescent endothelial cells (EC) have been identified in cardiovascular disease, in angiogenic tumour associated vessels and in aged individuals. We have previously identified a novel anti-inflammatory senescent phenotype of EC. We show here that caveolae are critical in the induction of this anti-inflammatory senescent state. Senescent EC induced by either the overexpression of ARHGAP18/SENEX or by H_2_O_2_ showed significantly increased numbers of caveolae and associated proteins Caveolin-1, cavin-1 and cavin-2. Depletion of these proteins by RNA interference decreased senescence induced by ARHGAP18 and by H_2_O_2_. ARHGAP18 overexpression induced a predominantly anti-inflammatory senescent population and depletion of the caveolae-associated proteins resulted in the preferential reduction in this senescent population as measured by neutrophil adhesion and adhesion protein expression after TNFα treatment. In confirmation, EC isolated from the aortas of CAV-1^*−/−*^ mice failed to induce this anti-inflammatory senescent cell population upon expression of ARHGAP18, whereas EC from wild-type mice showed a significant increase. NF-κB is one of the major transcription factors mediating the induction of E-selectin and VCAM-1 expression, adhesion molecules responsible for leucocyte attachment to EC. TNFα-induced activation of NF-κB was suppressed in ARHGAP18-induced senescent EC, and this inhibition was reversed by Caveolin-1 knock-down. Thus, out results demonstrate that an increase in caveolae and its component proteins in senescent ECs is associated with inhibition of the NF-kB signalling pathway and promotion of the anti-inflammatory senescent pathway.

## Introduction

Senescence plays a major role in many pathophysiological processes (Adams, 2009; Rodier & Campisi, 2010), including tumour suppression (Collado & Serrano, 2010), inhibition of liver damage (Krizhanovsky *et al*., 2008), and it is also linked to the progression of aging and age-related pathologies (Erusalimsky & Kurz, 2005). Further, recent work establishes senescence as important in development (Munoz-Espin *et al*., 2007; Storer *et al*., 2012). Senescence involves the permanent inhibition of cell cycle progression and the induction of a pattern of gene expression and secretion of factors referred to as the senescence associated secretory phenotype (SASP) (Coppe *et al*., 2010; Freund *et al*., 2010). The SASP factors are pro-inflammatory and can be beneficial, such as in the case of senescent hepatic stellate cells that limit hepatic fibrosis through secretion of matrix metalloproteinases (MMPs) (Krizhanovsky *et al*., 2008). In addition, SASP factors, such as IL-6 and IL-8, can act in an autocrine feedback loop to reinforce senescent growth arrest (Kuilman *et al*., 2008). In contrast, the SASP has also been found to have deleterious pathophysiological effects. Accumulation of senescent cells seen in aged mice may, through secretion of the SASP, contribute to low-level chronic inflammation, a phenotype common to age-related pathologies (Erusalimsky & Kurz, 2005; Minamino & Komuro, 1994; Freund *et al*., 2010). In support of this, Baker *et al*. demonstrated that specific inactivation of senescent cells in BubR1 progeroid mice can delay premature aging and age-related diseases (Baker *et al*., 2008).

In contrast to all other cell types in which the SASP is pro-inflammatory, we have shown that senescent endothelial cells (EC) can also acquire an anti-inflammatory phenotype. This phenotype is induced by three stimuli linked to aging (Coleman *et al*., 2013); oxidative stress, hypoxia and disturbed flow. Further, overexpression of the gene *ARHGAP18* or *SENEX* also induces a predominantly anti-inflammatory senescent phenotype as they fail to support neutrophil and mononuclear adhesion, have decreased E-selectin, VCAM-1 and IL-8 synthesis and are not responsive to the permeability inducing agent thrombin (Coleman *et al*., 2010). Recently, the Evans group has confirmed the induction of the anti-inflammatory EC senescent population with disturbed flow (Warboys *et al*., 2002). Thus, we hypothesise that this senescent cell type in the vasculature has an important role in limiting inflammation and disease progression.

Evidence that implicates caveolae as critical regulators of cellular senescence has been mounting over the last 10 years. Caveolae are 50–100 nm flask-shaped invaginations in the plasma membrane enriched in cholesterol, glycosphingolipids, the structural protein caveolin-1 (Cav-1) and adaptor proteins cavin-1 and cavin-2 (Chidlow & Sessa, 2010; Parton & del Pozo, 2009). Although originally suggested to function in endocytosis, potocytosis and cholesterol transport, caveolae play a critical role in cell signalling, regulation of lipid homeostasis, inflammation, tumour suppression and promotion of vascular tone and blood pressure [reviewed in (Parat, 2013)]. Cav-1 or cavin-1 overexpression in fibroblasts induces senescence through induction of the p53/p21^CIP1^ pathway (Fang *et al*., 2007; Bai *et al*., 2011; Volonte & Galbiati, 2012), whereas in cells harbouring antisense Cav-1 or fibroblasts from CAV-1 null mice, senescence is inhibited (Volonte *et al*., 2013; Bartholomew *et al*., 2009). Caveolae are abundant in EC populations and linked to mechanism of vaso-relaxation through shear or other stimulus-mediated NO production (Chai *et al*., 2013), and to inhibition of inflammation (Bucci *et al*., 2000). Here, we show that induction of the anti-inflammatory senescent phenotype in EC is specifically mediated through caveolae. ECs in which caveolae proteins were knocked down and ECs from CAV-1 knockout mice failed to develop the anti-inflammatory senescent phenotype following overexpression of ARHGAP18. The mechanism involves, at least in part, an inhibition of the NF-κB response. This study identifies a new mechanism explaining the mosaic of inflammatory responses developed by stress-induced senescent EC.

## Results

### Senescent ECs have increased caveolae and expression of Cav-1, cavin-1 and cavin-2

**Figure 1:**
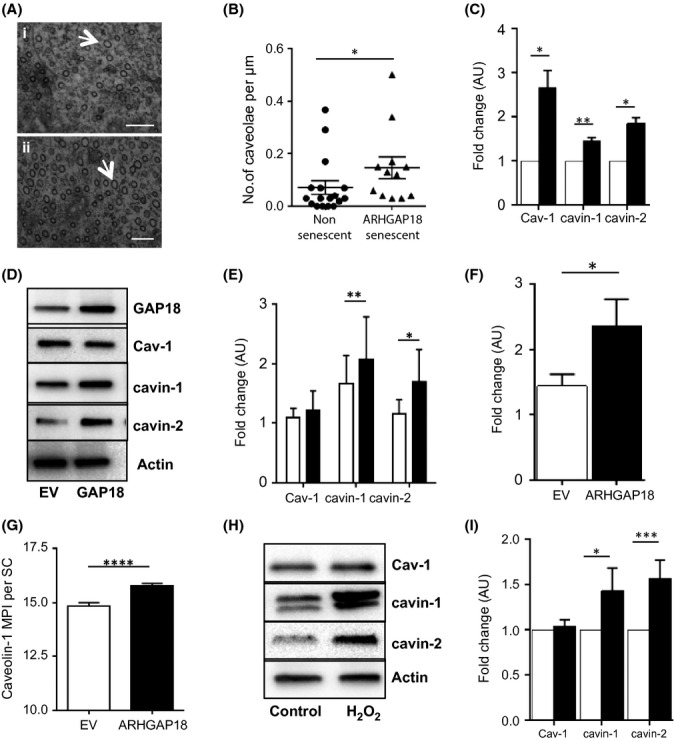
Upregulation of caveolae in ARHGAP18-induced senescent endothelial cells. (A–G) Human Umbilical Vein Endothelial Cells (HUVECs) were infected with EV (□) or *ARHGAP18* (■) containing adenovirus for 48 h. (A) Sections from cells classified as nonsenescent, basally induced senescent (i), or ARHGAP-18-induced senescent (ii) based on cross-sectional area were cut parallel to the substratum and analysed by electron microscopy. Arrows indicate a caveolae. (B) Sections were examined for the number of morphological caveolae per μm of linear surface. Only caveolae that were opened to exterior and of clear flask-shape structure were quantified. Data are the pool of three individual HUVEC lines, 12–15 cells in total where each point represents analysis of a single cell. Wilcoxon signed rank test was used based on a hypothetical value taken from the mean of nonsenescent cells, *P < 0.05. (C) Cells were harvested at 48 h, and mRNA levels determined. Mean ± SEM of four independent HUVEC lines with EV set to an arbitrary unit (AU) of 1.0. *P < 0.05; **P < 0.01 compared with EV. (D) Protein expression of ARHGAP18 (GAP18), Cav-1 (21–22 kDa), cavin-1, cavin-2 or β-actin as loading control was analysed. (E) Densitometry analysis of protein expression normalized to β-actin and untreated control. Results are mean ± SEM of expression relative to EV, 6–10 experiments using independent HUVEC lines; *P < 0.05, **P < 0.01. Data are expressed in arbitrary units (AU). (F) Cell lysates were probed for the high molecular weight (200 kDa) complex of Cav-1. Densitometry analysis of the protein expression normalized to β-actin and untreated control. Results are mean ± SEM of expression relative to EV, using eight independent HUVEC lines, *P < 0.05. Data expressed in arbitrary units (AU). (G) Cells were fixed, permeabilized and stained with DAPI. Senescent cells were analysed for expression of Cav-1 quantified by mean pixel intensity (MPI) using imagej. Images were taken randomly of 10 fields per line, data are mean of three independent HUVEC lines. Mean ± SEM, ****P < 0.0001. (H) Cells treated with 100 μm H_2_O_2_ for 48 h then lysed and analysed for Cav-1, cavin-1, cavin-2 or β-actin as loading control. (I) Densitometry analysis of control (□) or H_2_O_2_ (■)-treated cells normalized to β-actin. Control cells were set to an arbitrary unit (AU) of 1.0. Mean ± SEM, of 4–7 independent HUVEC lines, *P < 0.05, ***P < 0.001.

We have previously reported that ARHGAP18 (or SENEX) overexpression induced senescence in EC (ARHGAP18-senescent EC) (Coleman *et al*., 2010). Infection with an ARHGAP18-encoding adenovirus induced senescence in approximately 20% of the cells at 48 h post infection, as defined by the enlarged cell size, positive staining with SA-β-gal, p21 expression and loss of eNOS expression (Coleman *et al*., 2010, 2013). To investigate the mechanism underlying the induction of the anti-inflammatory phenotype, we first investigated the morphology of the enlarged ARHGAP18-senescent EC by electron microscopy (EM). A striking feature of these cells was the high density of caveolae in the senescent cells (Fig. 1A) similar to that seen previously in fibroblasts (Bai *et al*., 2011). Quantification from EM images based on only caveolae that had an opening to the exterior and had a flask-shaped morphology shows a significant increase in the number of caveolae in the ARHGAP18-senescent population (Fig. 1B). We confirmed this increase in caveolae in a number of ways. Firstly, at the mRNA level, there was increased expression of the key caveolae proteins Cav-1, cavin-1 and cavin-2 (Fig. 1C). Secondly, we find increased protein levels for cavin-1 and cavin-2 (Fig. 1D,E). No consistent change in 21–22 kDa form of Cav-1 was seen. However, there was a significant increase in the large 70S SDS-resistant Cav-1 complexes of apparent molecular weight of 200 kDa (Fig. S1A, 1F) that have been reported previously (Hayer *et al*., 2010). Thirdly, there was a significant increase in the level of phosphorylation on tyrosine 14 of Cav-1 (Fig. S1B). This phosphorylation is known to regulate recruitment of SH2 containing proteins (Lee et al. 2000) as well as the internalisation of lipid rafts and cell detachment (del Pozo et al. 2005). Fourthly, immunostaining for Cav-1 showed a significant increase in ARHGAP18-senescent cells compared with EV (empty vector) control cells (Fig. 1G). Increases in the caveolae-associated proteins cavin-1 and cavin-2 (Fig. 1H,I), as well as the high molecular weight complexes of Cav-1 (Fig. S1C,D), were also seen in H_2_O_2_-induced senescent EC. The doublet band seen in the Western blot for cavin-1 is seen in many but not all EC lysates and is similar to that reported in fibroblasts with increased passage number (Bai *et al*., 2011).Taken together, our data show that an upregulation of caveolae is a general feature of senescent EC rather than being restricted to ARHGAP18-induced senescent EC.

### Regulation of ARHGAP18-induced senescence by caveolae proteins

**Figure 2:**
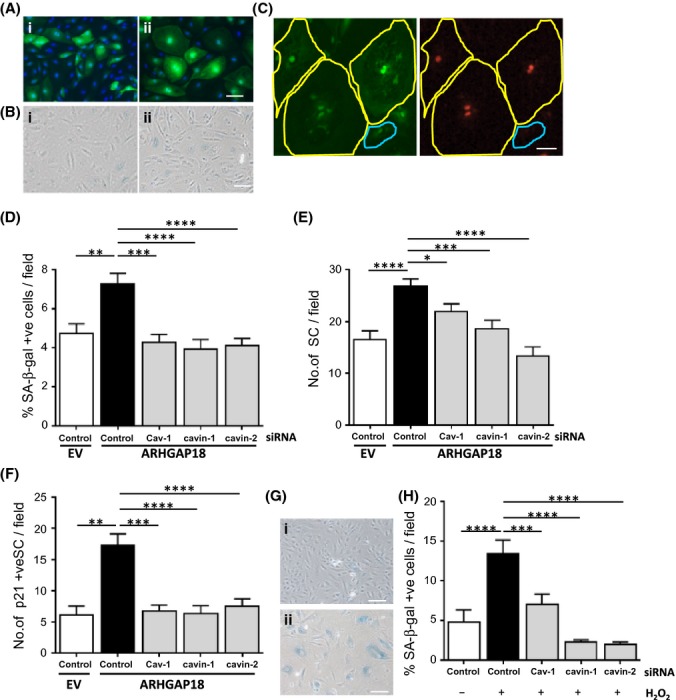
Knock-down of caveolae restrains endothelial cell (EC) senescence. (A–C) Human Umbilical Vein Endothelial Cells (HUVECs) were infected with EV- (i) or *ARHGAP18-* (ii) containing adenovirus for 48 h. (A) Morphology of GFP positive cells. Scale bar, 50 μm. (B) SA-β-gal stained cells. Scale bar, 100 μm. (C) Cells were stained for p21. Yellow circle indicates senescent cells and blue indicates nonsenescent cell. Scale bar, 25 μm. (D) HUVECs were transfected with siRNAs as indicated then infected with EV or ARHGAP18-containing adenovirus. After 48 h, cells were stained for SA-β-gal and the number of SA-β-gal positive, GFP positive, large senescent cells were counted in 10 random images per line from five separate HUVEC lines. Mean ± SEM, **P < 0.01, ***P < 0.001, ****P < 0.0001. (E) Cells prepared as from (D) were classified as senescent using large morphology and positive GFP expression. Cells in 10 random images per line, from eight separate HUVEC lines were counted. Mean ± SEM. *P < 0.05, ***P < 0.001, ****P < 0.0001. (F) Large p21 positive, GFP positive senescent cells from (C) were counted in five images per line from three separate HUVEC lines. Mean ± SEM, **P < 0.01, **P < 0.001, ****P < 0.0001. (G) HUVECs transfected with control siRNA or siRNA for the caveolae proteins were then untreated (i) or treated with 100 μm of H_2_O_2_ (ii) and stained with SA-β-gal. Scale bar, 100 μm. (H) Cells from (G) were classified as senescent and counted in 10 random images per line from three separate HUVEC lines. Mean ± SEM. ***P < 0.001, ****P < 0.0001.

After 48 h, ARHGAP18 overexpression resulted in a significant increase in large cells, defined as senescent based on size and flattened appearance (Fig. 2A), expression of SA-β-galactosidase (SA-β-gal) (Fig. 2B) and of p21 expression (Fig. 2C), as we have previously demonstrated (Coleman *et al*., 2010, 2013). These ARHGAP18-induced senescent ECs increase over time such that by 10 days, 70–80% are senescent. However, to investigate the early events in senescence induction, the 48-h time point was chosen for the subsequent experiments where we consistently see 10–20% of the cells have already become senescent.

To determine whether caveolae are important in the senescence induction, siRNA was used to deplete individual caveolae proteins. mRNA was depleted by >90% for all proteins (Fig. S2A), while the protein expression of Cav-1, cavin-1 and cavin-2 was reduced by >60% (Fig. S2B,C). Depletion of caveolae-associated proteins from the EV control population had no effect on the induction of senescence shown by SA-β-gal, morphology change or p21 expression (Fig. S3A–C). However, depletion of Cav-1, cavin-1 and cavin-2 reduced the number of ARHGAP18-senescent cells as judged by the decrease in the number of SA-β-gal positive cells (Fig. 2D), of enlarged flattened cells (Fig. 2E) and of p21 positive senescent cells (Fig. 2F). The depletion of the caveolae-associated proteins reversed the senescent-induced downregulation of eNOS (Fig. S4A,B). Further, the induction of H_2_O_2_-senescent EC (Fig. 2G) was also reversed by Cav-1, cavin-1 and cavin-2 depletion (Fig. 2H).

### Regulation of the inflammatory phenotype of ARHGAP18-induced senescent cells by caveolae

**Figure 3:**
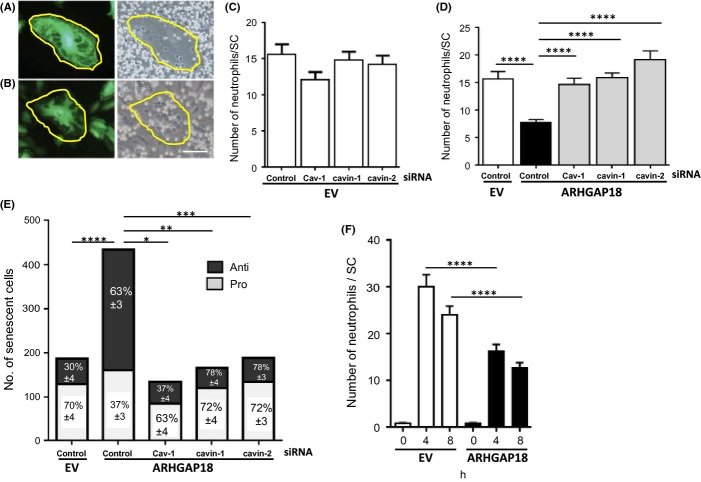
Cav-1, cavin-1 and cavin-2 are required to promote the anti-inflammatory phenotype of ARHGAP18-induced senescent cells. (A–E) Human Umbilical Vein Endothelial Cells (HUVECs) were treated with EV- or ARHGAP18-containing adenovirus for 48 h, stimulated with 5 ng mL^−1^ TNFα for 5 h then washed. Neutrophils were added for 1 h. Images were taken randomly of 10–12 fields per line. (A, B) Representative images of anti-inflammatory (A) and pro-inflammatory (B) senescent cell are circled. White neutrophils are adherent, and black neutrophils have transmigrated through endothelial cell (EC). Scale bar, 50 μm. (C, D) HUVECs were transfected with siRNA then treated with (C) EV-containing adenovirus or (D) ARHGAP18-containing adenovirus. The number of neutrophils that adhered to or transmigrated through senescent cells was counted. Mean ± SEM, data are mean of 3–7 independent HUVEC lines. ****P < 0.0001. (E) Senescent cells from the lines in (D) were classified as either pro- or anti-inflammatory based on the number of neutrophils adhered to, or transmigrated through the cell, compared to controls and expressed as a percentage. *P < 0.05, **P < 0.01, ***P < 0.001, ****P < 0.0001 is of the anti-inflammatory population. (F) HUVECs were treated with EV- or ARHGAP18-containing adenovirus for 48 h then stimulated with 5 ng mL^−1^ TNFα for 4 or 8 h, then washed. Neutrophils were added for 1 h. Images were taken randomly of 10 fields per line. The number of neutrophils adhered or transmigrated through senescent cells were counted. Mean ± SEM, data are mean of three independent HUVEC lines, ****P < 0.0001.

The inflammatory phenotype following TNFα stimulation was measured by neutrophil adhesion and adhesion molecule expression. Large flattened senescent cells were identified and then the extent of neutrophil adhesion to them was assessed. Cells that supported little to no neutrophil adhesion or transmigration were classified as anti-inflammatory (Fig. 3A), while those that showed neutrophil adhesion were classified as pro-inflammatory (Fig 3B). Following TNFα stimulation, EV control cells supported basal neutrophil adhesion (Fig. 3C, EV control) with approximately 70% showing pro-inflammatory and 30% anti-inflammatory characteristics (Fig. 3E, EV control). In ARHGAP18-senescent cells after TNFα stimulation, there was a decrease in the number of neutrophils that bound to the senescent cells (Fig. 3D, ARHGAP18 control) of which approximately 60% were judged to be anti-inflammatory and 40% of the senescent cells showed the normal pro-inflammatory response (Fig. 3E, ARHGAP18 control) and as we have shown previously (Coleman *et al*., 2010). The anti-inflammatory phenotype of the ARHGAP18-senescent cells was similar at both 4 and 8 h following TNFα stimulation (Fig. 3F) and with a similar proportion of anti-to pro-inflammatory cells (Fig. S5), suggesting that the anti-inflammatory phenotype is not due to delayed activation of the inflammatory response.

To determine the influence of caveolae proteins on the inflammatory phenotype, we depleted the caveolae proteins and investigated how this depletion affected the inflammatory phenotype. Depletion of the caveolae proteins in the EV cells did not significantly alter the basal level of neutrophil adhesion (Fig. 3C). However, depletion of the caveolae proteins reversed the lack of neutrophils adhering to the ARHGAP18-senescent cells, to levels seen in EV cells (Fig. 3D). When analysed at the individual cellular level for their inflammatory profile, the knock-down of all three caveolae proteins resulted in the preferential loss of the anti-inflammatory senescent cell phenotype and an increased acquisition of the pro-inflammatory senescent phenotype (Fig. 3E).

**Figure 4:**
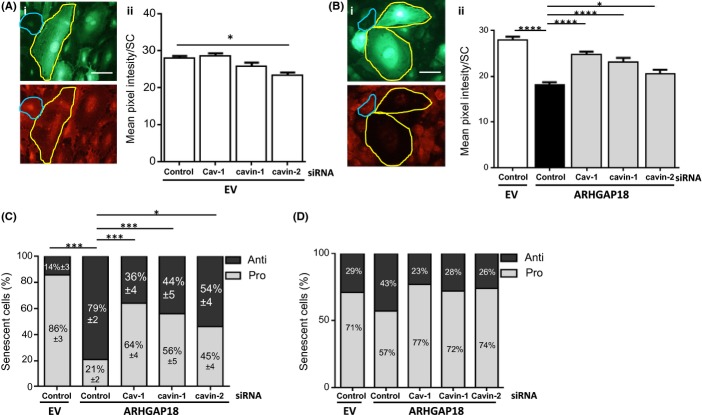
Cav-1, cavin-1 and cavin-2 are required to promote the anti-inflammatory phenotype of ARHGAP18-induced senescent cells via adhesion molecule expression. (A–D) Human Umbilical Vein Endothelial Cells (HUVECs) were transfected with control siRNA or siRNA against caveolae proteins then treated with EV- or ARHGAP18-containing adenovirus for 48 h and stimulated with 5 ng mL^−1^ TNFα for 5 h. (A, B) Cells were stained for DAPI and E-selectin. A representative EV (Ai) or ARHGAP18 (Bi) senescent cell is shown. Yellow indicates senescent cells and blue indicates nonsenescent cell. Scale bar, 50 μm. Senescent cells from EV- (Aii) or ARHGAP18- (Bii) overexpression were analysed for cell-surface expression of E-selectin and quantified by mean pixel intensity using imagej. Images were taken randomly of 10 fields per line, data are mean of 3–4 independent HUVEC lines. Mean ± SEM, *P < 0.05, ****P < 0.0001. (C) Senescent cells from (B) were classified as either pro- or anti-inflammatory based on E-selectin expression compared with controls and given as a percentage of the total population. Mean ± SEM of 3–4 independent HUVEC lines. *P < 0.05, ***P < 0.001. (D) Cells were stained for VCAM-1 expression. Senescent cells were analysed for cell-surface expression of VCAM-1 quantified by mean pixel intensity using Image J and then classified as pro- or anti-inflammatory based on VCAM-1 expression compared with controls and expressed as a percentage. Data is from one line, with two independent replicates and is representative of two experiments performed.

The importance of caveolae on the inflammatory phenotype of senescent cells was confirmed by the measurement of adhesion molecule expression. TNFα stimulation of EC induces neutrophil adhesion by upregulation of adhesion molecules such as E-selectin and VCAM-1 (Gamble *et al*., 1985). EV cells transfected with control siRNA and stimulated with TNFα showed the normal high levels of surface expression of E-selectin, either on normal (blue border) or basal senescent cells (yellow border) (Fig. 4Ai). Knock-down of the Cav-1 and cavin-1 proteins from EV cells had no significant effect, and knock-down of cavin-2 had only a minor change on E-selectin expression (Fig. 4Aii). In ARHGAP18-senescent cells, there was reduced E-selectin expression with control siRNA as visualized in Fig. 4Bi and quantified in Fig. 4Bii. Knock-down of Cav-1, cavin-1 or cavin-2 in these senescent cells showed a partial reversal of this, with higher levels of E-selectin surface expression (Fig. 4Bii). When the number of pro- and anti-inflammatory cells based on E-selectin expression after caveolae-associated protein knock-down was determined, there was a preferential decrease in the number of anti-inflammatory senescent cells, and a concomitant increase in the proportion of cells displaying the pro-inflammatory phenotype (Fig. 4C) similar to that seen for neutrophil adhesion. Similar reversal of the inflammatory phenotype was shown when VCAM-1 expression was analysed (Fig. 4D). In this case, however, the ratio of anti- to pro-inflammatory cells after ARHGAP18 overexpression was routinely 40:60, whereas it was approximately 70:30 when determined by E-selectin expression and neutrophil adhesion (Figs 3 and 4). Attempts to investigate the inflammatory phenotype of H_2_O_2_-induced senescent EC in the absence of caveolae proteins were unsuccessful as all cells underwent apoptosis after TNFα stimulation. This is consistent with the known protective role of caveolae against stress-induced apoptosis.

### ARHGAP18 restrains the SASP by inhibiting NF-κB and AP-1 activity

**Figure 5:**
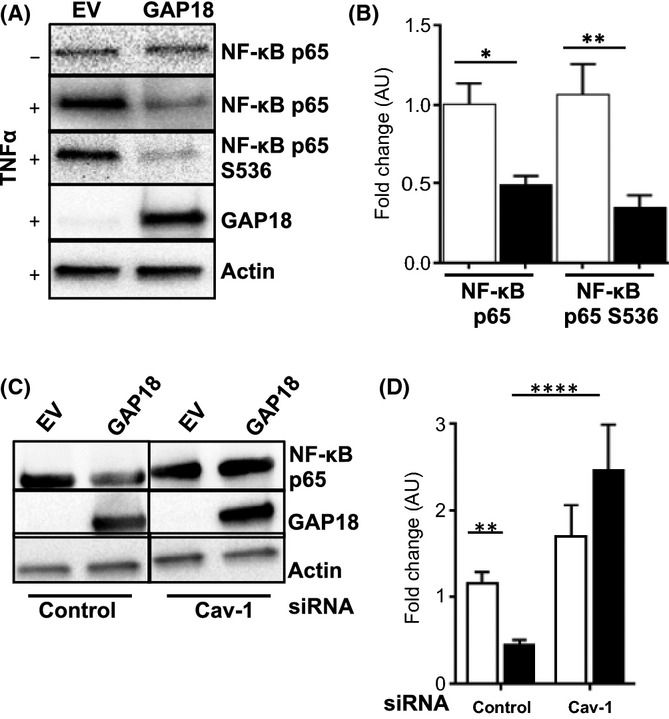
Caveolae regulate the anti-inflammatory phenotype in ARHGAP18-induced senescent cells through regulation of NF-κB. (A–D) Human Umbilical Vein Endothelial Cells (HUVECs) were infected with EV or ARHGAP18 (GAP18) for 48 h then stimulated with 5 ng mL^−1^ TNFα for 5 h (+) or unstimulated (−). (A) Total protein lysates were probed as indicated. (B) Densitometry analysis expressed in arbitrary units normalized to β-Actin and untreated control. EV (□) or ARHGAP18 (■). Mean ± SEM with EV set to an arbitrary unit (AU) of 1.0, of 4–6 independent HUVEC lines.*P < 0.05, **P < 0.01. (C) HUVECs were transfected with siRNA against Cav-1 then infected with an EV or ARHGAP18-containing adenovirus for 48 h. Lysate was probed for NF-κB p65, ARHGAP18 (GAP18) or β-Actin as loading control. Data is representative of a single HUVEC line. (D) Densitometry analysis expressed in arbitrary units normalized to β-Actin loading control and untreated control. EV (□) or ARHGAP18 (■). Mean ± SEM of four independent HUVEC lines; **P < 0.01, ****P < 0.0001.

NF-κB is a major transcription factor for inflammation, and in EC is essential for adhesion molecule expression (Lewis *et al*., 2000). In senescence, the NF-κB signalling pathway controls the generation of the SASP [reviewed in (Salminen *et al*., 2011)]. In the absence of TNFα stimulation, both EV and ARHGAP18-senescent cells showed basal levels of NF-κB expression (Fig. 5A). Following TNFα stimulation, EV cells showed increased expression of the RelA/p65 subunit of the NF-κB complex (Fig. 5A), whereas the ARHGAP18-senescent cells did not show this same upregulation (Fig. 5A,B). This lack of NF-κB activation was confirmed when the activation specific phosphorylation of S536 was measured (Fig. 5A,B). ARHGAP18-senescent cells also prevented the phosphorylation of c-Jun (Fig. S6A,B), an essential component of the transcription factor AP-1, which has also been shown to regulate components of the SASP and for adhesion molecule expression on EC (Salminen *et al*., 2011). Consistent with the importance of NF-κB in EC activation, depletion of Cav-1 resulted in a reversal in NF-κB levels (Fig. 5C,D).

### Expression of caveolin-1 is necessary for the anti-inflammatory phenotype

**Figure 6:**
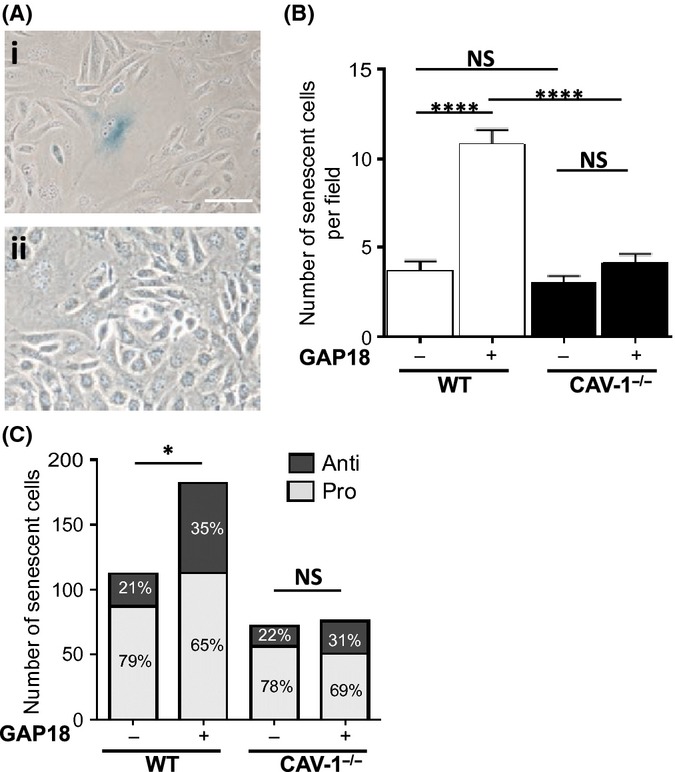
Cav-1 expression is required for premature senescence *ex vivo*. Aortic WT (i) and CAV-1^−/−^ (ii) ECs were treated with ARHGAP18 (GAP18)-containing adenovirus for 48 h then stained for SA-β-gal. Scale bar, 50 μm. (B) Cells, classified senescent by morphology, positive SA-β-gal staining and positive GFP expression were counted in 10–12 random fields per line from 3 to 4 independent isolates. WT (□) or CAV^−/−^ (■). ****P < 0.0001. (C) WT and CAV-1^−/−^ cells were treated with ARHGAP18-containing adenovirus for 48 h, 500 ng mL^−1^ TNF-α for 5 h, stained for VCAM-1 expression, quantified by MPI using imagej and classified as either pro- or anti-inflammatory. *P < 0.05 for comparison of the anti-inflammatory cells from six independent isolates per species.

The above results suggested that not only are caveolae critical to the induction of senescence, but in EC also to the anti-inflammatory phenotype. To confirm this, we examined the ability of EC from Caveolin-1 knockout mice to undergo senescence after ARHGAP18 overexpression. EC were outgrown from aortas explanted from CAV-1^−/−^ or WT littermates. The cells exhibited the classical cobblestone morphology and were confirmed to be EC by PECAM staining (data not shown). The WT ARHGAP18-infected ECs showed the morphology and SA-β-gal staining of senescent cells (Fig. 6Ai). In contrast, the majority of the CAV-1^−/−^ EC remained cobblestone, and very few SA-β-gal positive cells were seen following ARHGAP18 overexpression (Fig. 6Aii). Analysis of the number of senescent cells showed that both the WT and the CAV-1^−/−^ EC exhibited a similar low level of basal senescent cells, most likely replicative-induced senescent cells (Fig. 6B). Overexpression of ARHGAP18 in WT cells resulted in a significant increase in the number of senescent cells. In contrast, there was no significant increase in the number of senescent cells seen in the CAV-1^−/−^ EC (Fig. 6B). The inflammatory phenotype of the senescent cells was assessed by VCAM-1 expression following TNFα stimulation. The basal senescent cells seen in WT and CAV-1^−/−^ EC showed both the pro-inflammatory and anti-inflammatory senescent populations (Fig. 6C), with a similar proportion to that seen in human cells stained for VCAM-1 (Fig. 4D). WT cells overexpressing ARHGAP18 showed a significant increase in the number of anti-inflammatory senescent cells. In contrast, no significant increase in the senescent anti-inflammatory population was seen following ARHGAP18 overexpression in CAV-1^−/−^ ECs. As Cav-1 is essential for caveolae formation, this result confirms a role of caveolae in the induction of the anti-inflammatory phenotype in EC.

## Discussion

Endothelial senescence has been observed *in vivo* and *in vitro*, but the physiological relevance has chiefly been implied from the study of other cell types. Our demonstration, that senescent EC display an anti-inflammatory phenotype (Coleman *et al*., 2010, 2013) is thus unique and in stark contrast to other cell types described. Our results here demonstrate a molecular mechanism for the induction of this stress-induced anti-inflammatory phenotype mediated through upregulation of caveolae and the caveolae proteins and through the associated inhibition of NF-κB activation.

Although Cav-1 and cavin-1 have previously been linked to senescence induction in fibroblasts (Galbiati *et al*., 2001; Bai *et al*., 2011; Volonte & Galbiati, 2012), the role of these proteins in the inflammatory phenotype of senescent cells has not been investigated. Until recently, senescence has been defined as a pro-inflammatory process based on studies performed principally in fibroblasts, epithelial cells, keratinocytes and tumour cell lines. Our recent work has, however, demonstrated that senescent EC can acquire an anti-inflammatory phenotype (Coleman *et al*., 2010, 2013). This alternative phenotype can be induced by overexpressing the ARHGAP18 gene or as a result of oxidative stress, disturbed flow or hypoxia stimulation. ARHGAP18 overexpression has allowed us to dissect the involvement of caveolae in the development of the anti-inflammatory phenotype, and we have identified caveolae as critical regulators of the anti-inflammatory phenotype in senescent EC. This was demonstrated firstly by siRNA technology in which knock-down of any of the three caveolae proteins blocked the induced anti-inflammatory phenotype of senescent EC with a concomitant increase in the pro-inflammatory phenotype. The pro-inflammatory phenotype, that of increased neutrophil adhesion and induction of E selection and VCAM-1 expression following TNFα stimulation, is the response of normal nonsenescent EC to inflammatory cytokine activation. Thus, inhibition of ARHGAP18-mediated caveolae formation switches the cells from an anti-inflammatory back to their normal pro-inflammatory phenotype.

Secondly, the role of caveolae in regulating this novel anti-inflammatory senescent phenotype was confirmed using EC isolated from CAV-1^−/−^ aortas. Both WT and CAV-1^−/−^ EC displayed a small basal level of senescence, most likely as a result of replicative stress. Indeed, these basal senescent cells were predominantly pro-inflammatory although an anti-inflammatory population was also evident. ARHGAP18 overexpression in the CAV-1^−/−^ EC did not alter the number or profile of the senescent EC, in contrast to WT EC, in which there was a significant increase in number of senescent cells and in the anti-inflammatory population. Thus, the basal level of senescence in the CAV-1^−/−^ EC suggests that caveolae are not essential for all types of senescence induction in EC, as both pro-inflammatory and anti-inflammatory cells are seen in this un-induced population of senescent cells. However, caveolae are essential for the senescence induced by ARHGAP18 overexpression. This link of caveolae to the anti-inflammatory state is consistent with the work of Sessa and colleagues (Bauer *et al*., 2005) who showed, using EC specific transgenics, that an increase in Cav-1 results in a dampening of VEGF-induced permeability and angiogenesis, likely through regulation of eNOS. Indeed, ARHGAP18-induced senescence does result in a decrease in eNOS that is partially reversed by caveolae knock-down. Importantly, our results in the mouse aortic EC also show that the anti-inflammatory phenotype is a general phenotype seen in different EC types (veins and arteries) and also seen in human and mouse EC.

Although both oxidative stress and ARHGAP18 overexpression induce senescence, ARHGAP18 overexpression results specifically in an increase in the anti-inflammatory population. The mechanism underlying this unique feature of ARHGAP18 overexpression, to induce the anti-inflammatory senescent phenotype, remains to be elucidated but is independent of the GAP function of this protein (Coleman *et al*., 2010). Furthermore, the anti-inflammatory to pro-inflammatory phenotype of senescent cells is the same with a mutant ARHGAP18 as for the wild-type ARHGAP18 (Fig. S7). The ability of GAP proteins to have both GAP dependent and GAP independent roles is consistent with that seen in Drosophila for Conundrum, the Drosophila homologue of ARHGAP18 (Neisch *et al*., 2013). At present, we do not understand the mechanism behind the ARHGAP18-mediated upregulation of caveolae, except that our preliminary data have shown the importance of an ARHGAP18-associated protein, known to be involved in membrane curvature, in senescence induction.

A further observation from our studies shows that in EC and in contrast to that seen in fibroblasts, cavin-2 is also involved in senescence induction. The involvement of cavin-2 in senescence is not unexpected. Firstly, cavin-2 is known to induce quiescence in response to culture stress, demonstrating a role for cavin-2 in cell cycle regulation (Gustincich & Schneider, 1993). Secondly, Cav-1, cavin-1 and cavin-2 have been proposed to form a complex whereby expression of each member regulates expression of the other members and influences caveolae dynamics (Hansen *et al*., 2009). Although previous work has shown that siRNA depletion of cavin-2 in ECs ablates the number of caveolae, and significantly reduces the expression of the other caveolae proteins, cavin-2 is not essential for caveolae formation in all types of EC (Hansen *et al*., 2009, 2013; Bai *et al*., 2011).

The caveolae microdomain can promote pro- and anti-inflammatory signals likely through the localization of inflammatory receptors to the caveolae signalling platform (Chidlow & Sessa, 2010). As an anti-inflammatory mediator, Cav-1 has been shown to interact with COX-2 at the endoplasmic reticulum and enhance its degradation (Chen *et al*., 2010). Cav-1 has also been shown to inhibit the ERK1/2, JNK and PI(3)K pathways after integrin signalling (Engelman *et al*., 1998; Echarri & Del Pozo, 2006) and can act through p38 MAPK to inhibit the AP-1- and NF-κB-mediated pro-inflammatory responses (Wang *et al*., 2011). Cav-1 also inhibits HIV replication through inhibition of NF-κB acetylation (Simmons *et al*., 2008). Indeed, in our ARHGAP18 driven senescence induction in EC, we show an inhibition of NF-κB activation and this is reversed by caveolae protein knock-down. Given the importance of NF-κB signalling in EC activation and promotion of the pro-inflammatory state (Kempe *et al*., 2005), we would suggest that caveolae regulation of NF-κB is an important fulcrum for control of inflammation. Indeed, Pavlides *et al*. (2013) have shown that CAV-1^−/−^ mice have increased inflammation due to increased HIF and NF-κB signalling (Pavlides *et al*., 2013). Furthermore, low Cav-1 expression correlates with high IL-8, high MMP-9, high IL-6 and general plaque vulnerability in atherosclerotic plaques (Rodriguez-Feo *et al*., 2005) demonstrating a potential anti-inflammatory pathophysiological role of Cav-1.

Endothelial cells are essential regulators of inflammation, defining the magnitude and timing of inflammation (Gamble *et al*., 1985). Upon activation by inflammatory cytokines, not only do EC induce a spectrum of pro-inflammatory activators, but there also occurs induction of negative regulators, such as TGF-β and PAI-1 in a self-regulatory loop to limit or inhibit the activation status (Gamble & Vadas, 1988; van Hinsbergh *et al*., 1988). Thus, the induction of an anti-inflammatory senescent population of EC, together with senescent cells that display the classic pro-inflammatory state would be in keeping with this concept. We propose that the anti-inflammatory senescence state in EC is a major pathway for inhibition of disease. Thus, in situations of high or chronic insult, senescence protects the endothelium from apoptosis, thus maintaining the essential coverage of the underlying tissue. In addition, the anti-inflammatory senescent phenotype would act in an attempt to inhibit or reverse the inflammatory response and thus restore the vasculature to homeostasis. The molecular characterization and the ability to identify these anti-inflammatory senescent cells *in vivo* will be important in confirming this possibility. However, given the scarcity of senescent-specific markers that can be used *in vivo*, and the difficulty of investigating individual EC phenotypes *in vivo* after activation, this is proving to be a major tour de force.

## Materials and methods

### Cell culture

Human Umbilical Vein Endothelial Cells (HUVECs) of Human Coronary Artery Endothelial Cells (HCAECs) were maintained in a 5% CO_2_ atmosphere under subconfluent conditions at all times and passaged every 2–3 days. Cells were lifted using 0.5% (w/v) trypsin and between 3 × 10^4^ and 8 × 10^5^ were passaged on either Lab-Tek 8 Chamber Slides (Thermo Fischer Scientific Inc., MA, USA), 25 cm^2^ flasks or 75 cm^2^ flasks.

### Adenovirus production and generation of HUVEC overexpressing ARHGAP18

Adenovirus production was performed as previously described (Coleman *et al*., 2010) and is listed in Appendix S1 (Supporting information).

### siRNA transfection

Details of siRNA transfection are given in Appendix S1 (Supporting information).

### β-galactosidase activity

Human Umbilical Vein Endothelial Cells were fixed in 2% formaldehyde/0.2% glutaraldehyde in phosphate-buffered saline (PBS) for 15 min. Cells were incubated at 37 °C for 36–60 h in complete staining solution [2 mm MgCl_2_, 0.02% Nonident®- P-40, 0.01% sodium deoxycholate, 40 mm sodium citrate, 150 mm NaCl, 5 mm potassium ferrocyanide, 5 mm potassium ferricyanide, 1 mg mL^−1^ X-gal (Calbiochem, Nerck, Darmstadt, Germany) dissolved in 20 mg mL^−1^ dimethylformaide, pH 6.0]. Images were taken on the Eclipse Ti-*U* microscope (Nikon Instruments Inc., NY, USA).

### Antibodies

Primary antibodies are listed in Appendix S1 (Supporting information).

### Immunofluorescence

Human Umbilical Vein Endothelial Cells were plated onto Lab-Tek 8-well chamber slides (Thermo Fischer Scientific) pre-coated with 20 μg mL^−1^ Fibronectin. Details of immunostaining are listed in Appendix S1 (Supporting information).

### Western blot analysis

Immunoblotting experiments performed as given in Appendix S1 (Supporting information).

### Relative quantitative reverse transcription polymerase chain reaction

Total RNA was extracted from HUVECs using TRIzol® reagent (Invitrogen, Life Technologies Co., NY, USA) according to the manufacturer's instructions. Further details are given in Appendix S1 (Supporting information).

### Neutrophil cell isolation

Neutrophils were prepared from fresh blood donated by healthy donors. Blood was dextran sedimented, and cells were separated by Histopaque (Sigma-Aldrich Co. LLC., MO, USA) gradient centrifugation. Neutrophils were purified from the cell pellet with hypotonic lysis of the remaining red blood cells.

### Neutrophil adhesion assay

Human Umbilical Vein Endothelial Cells were plated on gelatine-coated six-well plates at 6 × 10^4^ for 2 h. At 50% confluence, monolayers were treated with TNFα for 5 h then washed in PBS. Freshly extracted neutrophils were added to the HUVECs at 6 × 10^6^ per well of a six-well plate. After 1 h, cells were thoroughly washed and photographs were taken using the Eclipse Ti-*U* microscope. Number of neutrophils adhered to, or transmigrated through, HUVECs were counted.

### Aortic explants from mice

Details of the aortic EC explants from mice are given in Appendix S1 (Supporting information).

### Statistics

Statistical analysis was performed using GRAPHPAD PRISM version 5.04 for Windows (GraphPad Software Inc., CA, USA) Windows. For direct comparison between two groups, statistical significance was calculated using unpaired student's *t*-test unless otherwise stated. Analysis of variance between three or more groups was measured using one-way anova with Tukey's or Dunnett's post-test, where appropriate. *P* values that were equal to or below 0.05 were considered significant: *denotes *P* < 0.05, **P < 0.01, ***P < 0.001, ****P < 0.0001. Data are presented as mean ± SEM.

## Supporting Information

**Fig. S1** Cav-1 expression in HUVECs. **Fig. S2** Depletion of caveolae proteins by RNA interference. **Fig. S3** Depletion of caveolae does not affect number of basally-induced senescent ECs. **Fig. S4** Caveolae protein depletion promotes eNOS expression in senescent cells. **Fig. S5** ARHGAP18 induced inflammatory phenotype is stable. **Fig. S6** Caveolae regulate the anti-inflammatory phenotype of ARHGAP18-induced senescent cells through regulation of c-Jun. **Fig. S7** The RhoGAP domain of ARHGAP18 is not responsible for the anti-inflammatory profile of senescent ECs.
