# Supplementary material for: Caveolae control the anti-inflammatory phenotype of senescent endothelial cells
Source: Aging Cell. 2014 Nov 19;14(1):102–11. doi: 10.1111/acel.12270 (PMC4326911; doi:10.1111/acel.12270)

Supplementary Figure 1

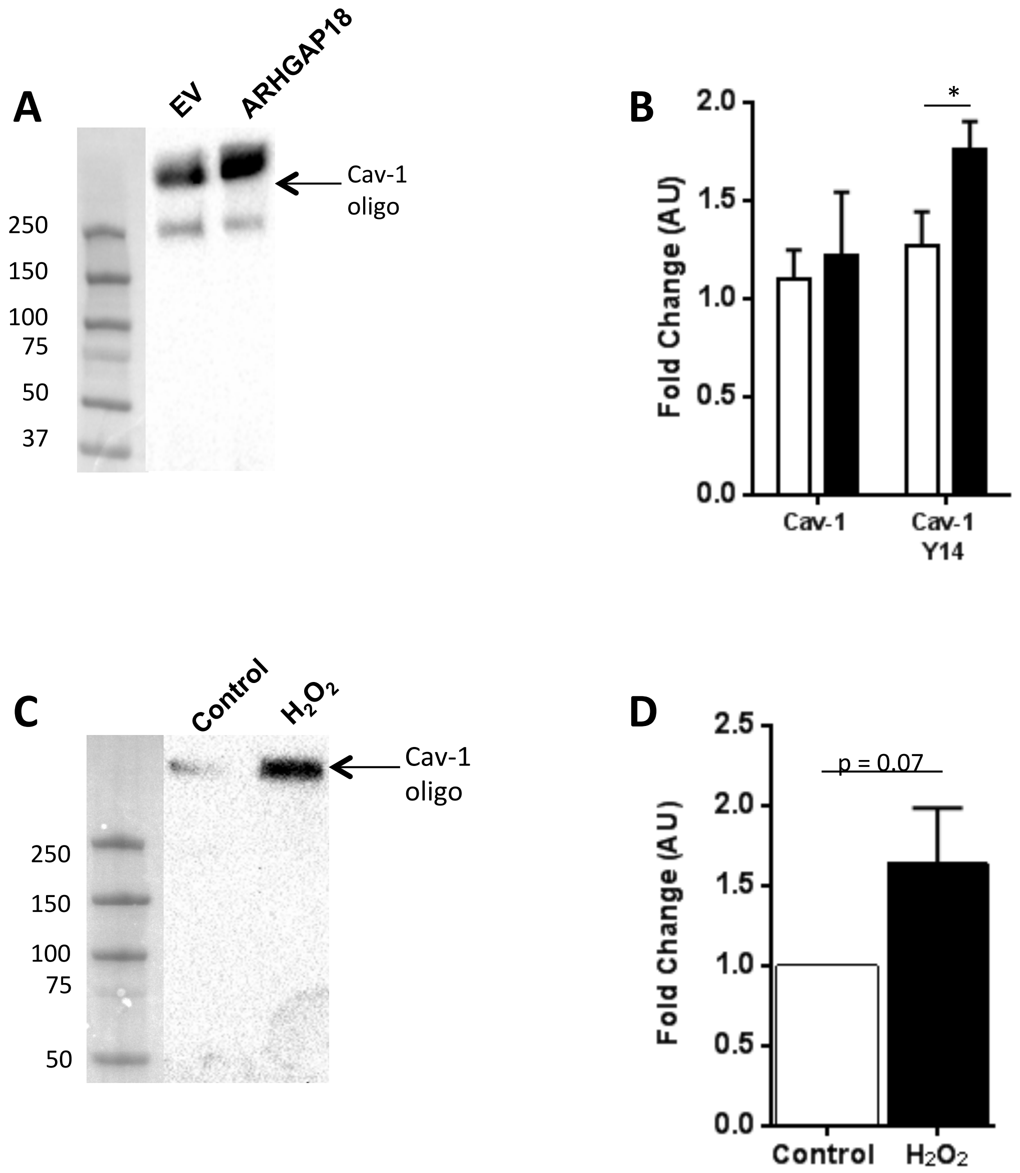

Supplementary Figure 2

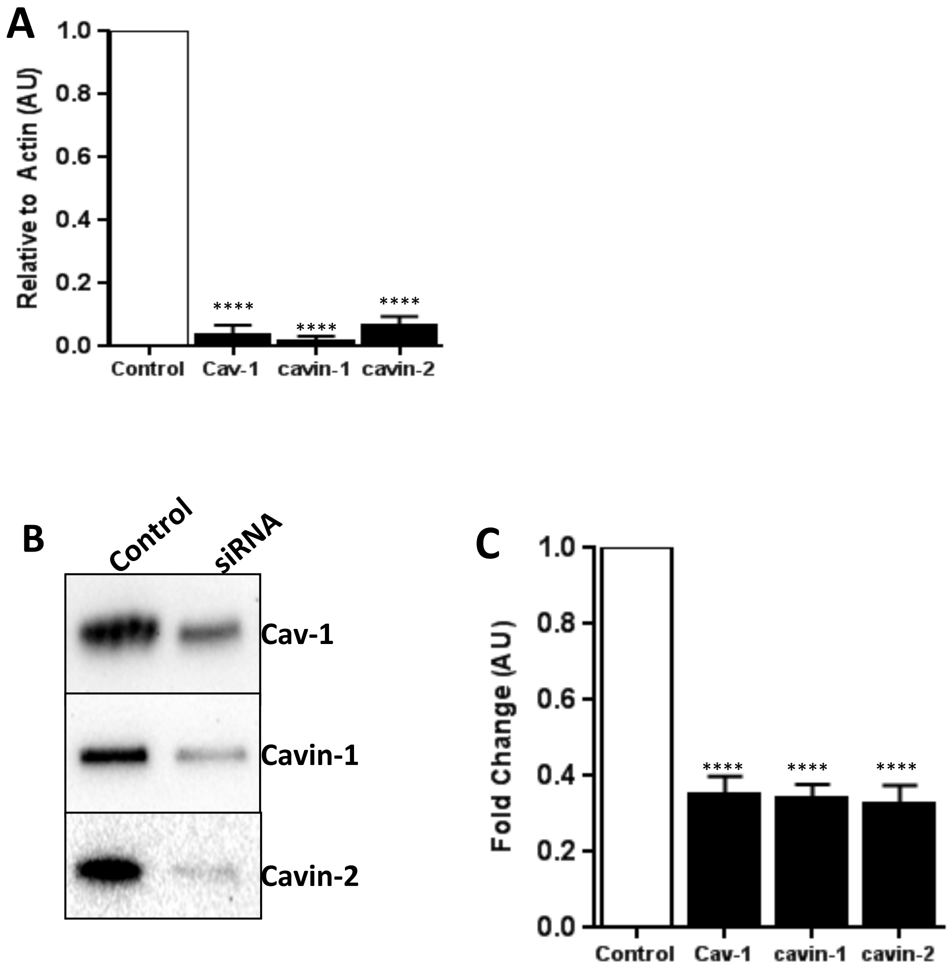

Supplementary Figure 3

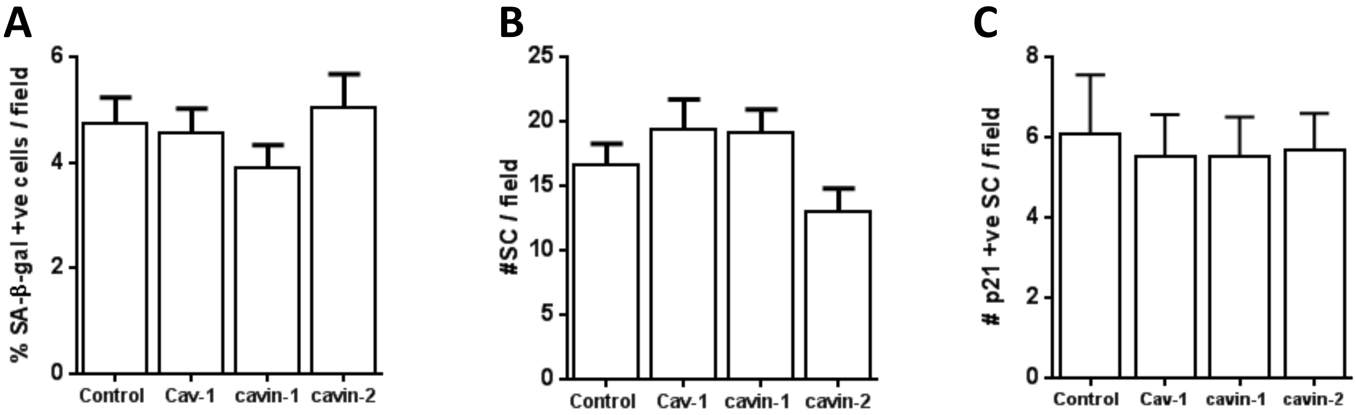

Supplementary Figure 4

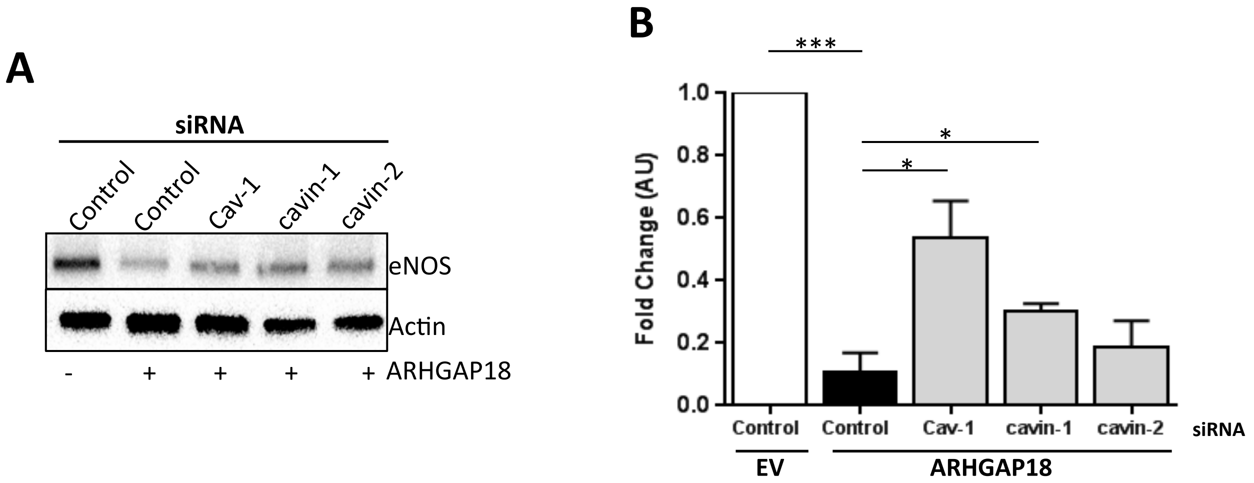

Supplementary Figure 5

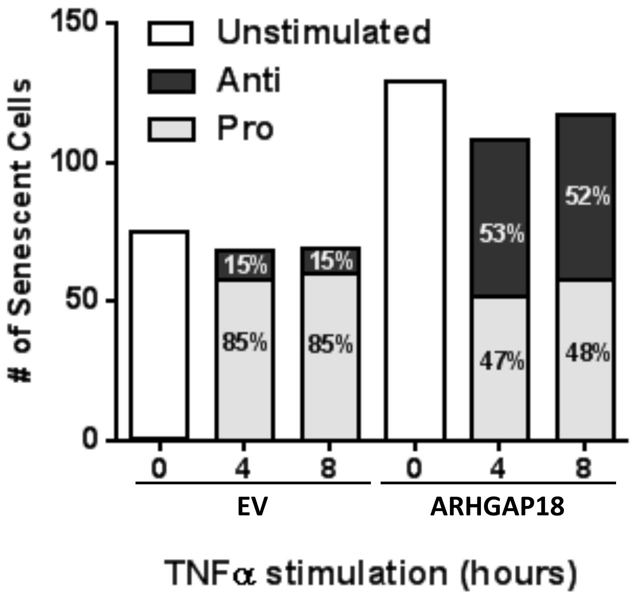

Supplementary Figure 6

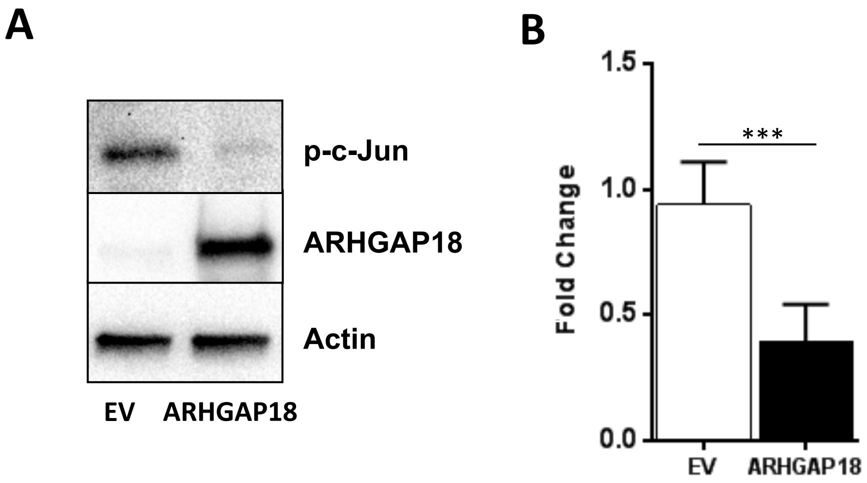

Supplementary Figure 7

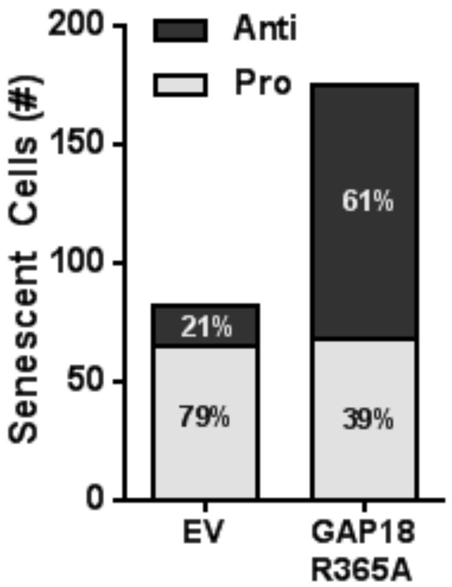

Supplement: Supplementary file 1 [file acel0014-0102-sd1.pdf]
