# Supplementary material for: Caveolae control the anti-inflammatory phenotype of senescent endothelial cells
Source: Aging Cell. 2014 Nov 19;14(1):102–11. doi: 10.1111/acel.12270 (PMC4326911; doi:10.1111/acel.12270)
Supplement: Supplementary file 2 [file acel0014-0102-sd2.docx]

**Supplementary Figure Legends:**

**Supplementary Figure 1: Cav-1 expression in HUVECs.**

**(A)** HUVECs were infected with EV- or ARHGAP18-containing adenovirus or left untreated. 48 hours later cells were lysed and probed for Cav-1. **(B)** HUVECs were infected with EV- or ARHGAP18 containing adenovirus for 48 hours then lysed and probed for Cav-1 or Cav-1 Y14. Densitometry analysis of protein expression from EV (□) or ARHGAP18 (■) infected cells normalised to β-actin and untreated control. Results are mean ± SEM of expression relative to EV expressed in arbitrary units (AU), using 3-9 independent HUVEC lines; unpaired two tailed student’s t-test was used, *p<0.05. **(C)** HUVECs were treated with 100μM H_2_O_2_ or left untreated. 48 hours later cells were lysed and probed for Cav-1. **(D)** Densitometry analysis of the oligos from **(C)** of protein expression normalised to β-actin and untreated control. Results are mean ± SEM of expression relative to EV expressed in arbitrary units (AU), using 8 independent HUVEC lines; unpaired two tailed student’s t-test was used.

**Supplementary Figure 2: Depletion of caveolae proteins by RNA interference.**

**(A-C)** HUVECs were transfected with siRNA targeting *Cav-1, cavin-1* or *cavin-2* or a scrambled control siRNA. **(A)** Total mRNA was extracted at 48 hours and knockdown confirmed by qPCR. Results are the relative decrease compared to β-actin expressed in arbitrary units (AU). Data represent 7 independent HUVEC lines, results are mean ± SEM. One way ANOVA with Dunnet post-test was used, ****p < 0.0001. **(B)** Western blot of caveolae protein expression after siRNA knockdown. **(C)** Densitometry analysis of **(B)** presented as Cav-1, cavin-1 and cavin-2 expression normalised to β-actin and control. Bars are mean ± SEM, of 3 independent HUVEC lines expressed in arbitrary units (AU). Paired two-tailed student’s t-test was used, ****p < 0.0001.

**Supplementary Figure 3: Depletion of caveolae does not affect number of basally-induced senescent ECs.**

**(A-C)** HUVECs were transfected with siRNAs as indicated then infected with EV-containing adenovrius. **(A)** After 48 hours cells were stained for SA-β-gal and the number of SA-β-gal positive, GFP positive, large senescent cells were counted in 10 random images per line from 5 separate HUVEC lines. **(B)** Cells prepared as in **(A)** were classified as senescent using large morphology and positive GFP. Cells in 10 random images per line, from 8 separate HUVEC lines were counted. **(C)** After 48 hours, cells were fixed and stained for DAPI and p21. Large p21 positive, senescent cells were counted in 5 images per line from 3 separate HUVEC lines.

**Supplementary Figure 4: Caveolae protein depletion promotes eNOS expression in senescent cells.**

**(A)** HUVECs were transfected with siRNA and then 4 hours later infected with EV- or ARHGAP18-containing adenovirus. 48 hours later cells were analysed for eNOS and β-actin as loading control. **(B)** Densitometry analysis of **(A)** presented as eNOS expression normalised to β-actin and EV set to an arbitrary unit of 1.0. Mean ± SEM. Pool of 4 independent HUVEC isolates. Unpaired two-tailed student’s t-test was used; * p < 0.05, *** p < 0.001.

**Supplementary Figure 5: ARHGAP18 induced inflammatory phenotype is stable.**

HUVECs were treated with EV- or ARHGAP18-containing adenovirus for 48 hours then stimulated with 5 ng/mL TNFα for 4 or 8 hours or left unstimulated (white bar), and then washed. Neutrophils were added for 1 hour. Images were taken randomly of 10 fields per line. The number of neutrophils adhered to, or transmigrated through, senescent cells were counted. Senescent cells were classified as either pro- or anti-inflammatory based on the number of neutrophils adhered to, or transmigrated through the cell, compared to controls and expressed as a percentage. Data is mean of 3 independent HUVEC lines.

**Supplementary Figure 6: Caveolae regulate the anti-inflammatory phenotype of ARHGAP18-induced senescent cells through regulation of c-Jun.**

HUVECs were treated with EV- or ARHGAP18-containing adenovirus for 48 hours then were stimulated with 5 ng/mL TNFα for 5 hours. **(A)** Total protein lysates were subjected to gel electrophoresis and probed for antibodies against phospho-c-Jun, ARHGAP18 or β-Actin (loading control). **(B)** Densitometry analysis of protein band intensity expressed in arbitrary units. Data is presented as protein expression normalised to β-Actin and untreated control, relative to EV control expressed in arbitrary units (AU). Bars are mean ± SEM, data is representative of 4-6 independent HUVEC lines. Paired two-tailed student’s t-test was used, *p <0.001.

**Supplementary Figure 7: The RhoGAP domain of ARHGAP18 is not responsible for the anti-inflammatory profile of senescent ECs.**

HUVECs were infected with EV- or ARHGAP18-R365A mutant construct containing adenovirus. After 48 hours cells were treated with 5ng/mL TNFα for 5 hours and washed. Neutrophils were added for 1 hour. Senescent cells were classified as either pro- or anti-inflammatory based on the number of neutrophils adhered to, or transmigrated through, the cell compared to controls and expressed as a percentage. Data is of three individual HUVEC lines.

**Supplementary Methods:**

*Adenovirus production and generation of HUVEC overexpressing* ARHGAP18:

The AdEasy system (Obigene) was used to produce recombinant adenovirus carrying human *ARHGAP18* and green fluorescent protein (GFP) or emptry vector (EV) according to the Qbiogene Version 1.4 AdEasy Vector system manual. Equivalent plaque forming units (pfu) per cell were adjusted to yield a similar level of GFP expression as determined by flow cytometry.

*siRNA Transfection:*

HUVECs were transfected with validated Stealth siRNAs (Invitrogen). *Caveolin-1* (HSS141466, HSS141467, HSS141468)(10nM), *Cavin-1* (HSS138488, HSS138489, HSS178652)(10nM) and *Cavin-2* (HSS112264, HSS112265, HSS112266)(20nM) were used to knockdown Caveolin-1, Cavin-1 and Cavin-2, respectively in parallel with corresponding nonspecific Stealth siRNA negative control (Invitrogen). Transfection of siRNA duplexes was performed using the Lipofectamine RNAIMAX Transfection Reaent (Invitrogen) according the manufacturer’s protocol.

*𝛽-galactosidase activity:*

HUVECs were fixed in 2% formaldehyde/0.2% glutaraldehyde in phosphate buffered saline (PBS) for 15 minutes. Cells were incubated at 37°C for 36-60 hours in complete staining solution (2 mM MgCl_2_, 0.02% Nonident®- P-40, 0.01% sodium deoxycholate, 40 mM sodium citrate, 150 mM NaCl, 5 mM potassium ferrocyanide, 5 mM potassium ferricyanide, 1 mg/mL X-gal [Calbiochem] dissolved in 20 mg/mL dimethylformaide, pH 6.0). Images were taken on the Eclipse Ti-*U* microscope (Nikon).

*Antibodies:*

Primary antibodies were from Cell Signalling Tech (Caveolin-1, 3238, 1:5000; NF-κB p65, 4764, 1:1000; NF-κB p65 S536, 3033, 1:1000; P-c-Jun, 9261, 1:1000), Proteintech (Cavin-1, 18892-1-AP, 1:5000; Cavin-2, 12339-1-AP, 1:2000), BD Transduction Laboratories (eNOS, 610297, 1:2000), Abcam (Caveolin-1 Y14, ab75876, 1:1000). The antibody to ARHGAP18 was raised as described previously (Coleman, et al).

*Immunofluorescence:*

HUVECs were plated onto Lab-Tek 8-well chamber slides (Thermo Fischer Scientific) pre-coated with 20 μg/mL Fibronectin. Where indicated, monolayers cultured on the slides were pre-incubated with TNF-α (5 ng/mL) for 4-8 hours before the assay depending on experiment, and then washed. Cells were fixed with 4% paraformaldehyde in Dulbecco’s phosphate buffered saline (DPBS), permeabilised in 0.1% Triton X-100 and blocked in 2 % bovine serum albumin with 0.5% goat serum. Primary antibodies were hybridised for 1 hour and binding detected by incubation with Alexa 594 fluorophore-coupled secondary antibody (Invitrogen) with DAPI (Sigma-Aldrich). Images were taken on the Leica TCS SP5 Confocal Microscope (Leica Microsystems).

*Western blot analysis:*

HUVEC lysates were prepared in ice-cold lysis buffer (50 mM Tris-HCl, pH 7.4 with 1% NP-40, 150 mM NaCl, 2 mM EDTA, 100 mM NaF, 10 mM sodium pyrophosphate and protease inhibitor cocktail (Sigma-Aldrich) and phosphatise inhibitor (Roche). Total protein was measured using BCA kit, and equal amounts of protein were loaded onto 4-15% Mini-PROTEAN ® TGX^TM^ Precast Gel (Bio-Rad), separated by SDS-PAGE, transferred to a polyvinylidene difluoride (PVDF) membrane and blocked with 5% skim milk powder and 0.1% Tween20 in PBS. Membrane was incubated with primary antibodies and after washing, membranes were incubated with secondary goat anti-rabbit or anti-mouse antibody conjugated to horseradish peroxidise and reactive bands detected by chemiluminescence (ECL Western Blotting Detection Reagents, Thermo Fischer Scientific).

*Relative quantitative reverse transcription polymerase chain reaction:*

Total RNA was extracted from HUVECs using TRIzol® reagent (Invitrogen) according to the manufacturer’s instructions. Two μg of DNase I treated (Sigma-Aldrich) RNA was reverse transcribed in a final volume of 20 μl using a High-Capacity cDNA Reverse Transcription Kit (Applied Biosciences) according to the manufacturer’s instructions and cDNA was used for subsequent quantitative PCR. The primers used for PCR were as follows: *ARHGAP18* (forward: 5’- ATGAGCTGGCTCTCCAGTT-3’; reverse: 5’- CTACAATGGCTTTGACTTTATAA), *Caveolin-1* (forward: 5’- TCTCTACACCGTTCCCATCC-3’; reverse: 5’- CAGGTCGATCTCCTTGGTGT-3’), *Cavin-1* (forward: 5’- AAGAAGCTGGAGGTCAACGA-3’; reverse: 5’- TCCGACTCTTTCAGCGACCC-3’), *Cavin-2* (forward: 5’- CCAAATGACCAGGAAGAGGA-3’; reverse: 5’- CGCGTAGCTACCCTCATAGC-3’). The threshold cycle values for all genes were normalised to the threshold cycle values determined for *𝛽-actin*, which remained unaltered during treatments. All PCR assay were repeated at least three times from independent RNA preparations.

*Aortic Explants from Mice:*

Cav-1^-/-^ gene knockout mice were fed standard chow ad libitum for 8 weeks. After bleeding, mice were perfused with cold phosphate-buffered saline. The aortas (thoratic and abdominal) were cleaned of extraneous fat, excised (above the super-renal aorta) and placed in ice-cold 10 x RPMI media (Sigma). Aorta was sectioned into ~1 mm rectangular pieces and embedded in a collagen gel. The collagen solution was obtained by mixing 7.5 volumes of 2 mg/mL rat tail type I collagen (BD Biosciences) with 1.5 volumes of 2.34 mg/mL NaHCO_2_ and 1 volume of 10 x RPMI medium (Gibco, Life Technologies) and ~0.1 volumes of 1M NaOH to adjust pH to 7.4. After 4 hours, growth media was added. The growth media solution contained 1 x RPMI supplemented with 10% Hyclone foetal bovine serum (Thermo Scientific), glutamine, 200U/mL gentamicin, 100 U/mL penicillin and 100 μg/mL streptomycin. The aortic explants were cultured at 37°C, 5% CO_2_ and after 7-10 days, explants were treated with collagenase to disperse monolayer cells and plated onto gelatine coated 25cm^2^ plates in growth media solution. After 6 days, endothelial cells were purified using magnetic-activated cell sorting (MACS) according to the manufacturer’s instructions. In brief, cells were harvested, counted, washed in sterile MACS buffer and resuspended in mouse FcR Blocking Reagent (1:10; Miltenyi Biotec). After 10 minutes, cells were washed and resuspended in purified rat-anti mouse CD31, rat anti-mouse CD105 and rat anti-mouse CD106 (BD Pharminogen) and incubated at 4°C for 30 minutes. After washing, cells were resuspended in anti-rat IgG Microbeads (Miltenyi Biotec) and incubated 4°C for 15 minutes. Cells were then washed and cell suspension was applied to an LS Column. LS Column was removed from the MACS separator and CD31+, CD105+, CD106+ cells were flushed out with MACS buffer. Labelled cells were counted and plated onto gelatine coated 25cm^2^ plates in growth media solution.
